# Ammonium salicylate: a synchrotron study

**DOI:** 10.1107/S1600536809029857

**Published:** 2009-08-08

**Authors:** Jae-Hyun Park Klepeis, William J. Evans, Natalia Zaitseva, Eric Schwegler, Simon J. Teat

**Affiliations:** a Lawrence Livermore National Laboratory, 7000 East Avenue, Livermore, CA 94550, USA; b Advanced Light Source, UC Berkeley, 1 Synchrotron Road, Berkeley, CA 94720, USA

## Abstract

The structure of the title salt, NH_4_^+^·C_7_H_5_O_3_^−^, is stabilized by substantial hydrogen bonding between ammonium cations and salicylate anions that links the components into a two-dimensional array.

## Related literature

For background to organic scintillators, see: Brooks (1979 ▶); Kaschuck *et al.* (2002 ▶); Kachuk & Esposito (2005 ▶). For the structures of salicylate salts, see: Wiesbrock & Schmidbaur (2003*a* ▶,*b* ▶); Dinnebier *et al.* (2002 ▶). For hydrogen bonding in salicylate compounds, see: Gellert & Hsu (1983 ▶); Drake *et al.* (1993 ▶).
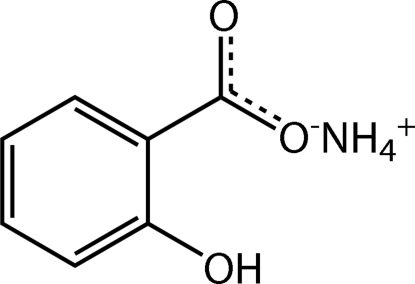


## Experimental

### 

#### Crystal data

NH_4_^+^·C_7_H_5_O_3_^−^
*M* *_r_* = 155.15
Monoclinic, 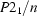

*a* = 6.0768 (6) Å
*b* = 20.089 (2) Å
*c* = 6.3353 (7) Å
β = 102.768 (1)°
*V* = 754.28 (14) Å^3^
*Z* = 4
Synchrotron radiation
λ = 0.77490 Å
μ = 0.13 mm^−1^
*T* = 150 K
0.40 × 0.20 × 0.06 mm

#### Data collection

Bruker APEXII diffractometer
Absorption correction: multi-scan (*SADABS*; Sheldrick, 2004 ▶) *T*_min_ = 0.950, *T*_max_ = 0.992
7758 measured reflections
2274 independent reflections
1939 reflections with *I* > 2σ(*I*)
*R*_int_ = 0.053

#### Refinement

*R*[*F* ^2^ > 2σ(*F* ^2^)] = 0.049
*wR*(*F* ^2^) = 0.150
*S* = 1.10
2274 reflections
136 parameters
All H-atom parameters refined
Δρ_max_ = 0.36 e Å^−3^
Δρ_min_ = −0.24 e Å^−3^

### 

Data collection: *APEX2* (Bruker, 2006 ▶); cell refinement: *SAINT* (Bruker, 2006 ▶); data reduction: *SAINT*; program(s) used to solve structure: *SIR97* (Altomare *et al.*, 1999 ▶); program(s) used to refine structure: *SHELXL97* (Sheldrick, 2008 ▶); molecular graphics: *ORTEP-3* (Farrugia, 1997 ▶); software used to prepare material for publication: *SHELXL97*.

## Supplementary Material

Crystal structure: contains datablocks global, I. DOI: 10.1107/S1600536809029857/tk2513sup1.cif

Structure factors: contains datablocks I. DOI: 10.1107/S1600536809029857/tk2513Isup2.hkl

Additional supplementary materials:  crystallographic information; 3D view; checkCIF report

## Figures and Tables

**Table 1:** Hydrogen-bond geometry (Å, °)

*D*—H⋯*A*	*D*—H	H⋯*A*	*D*⋯*A*	*D*—H⋯*A*
O1—H1⋯O3	0.93 (2)	1.66 (2)	2.523 (1)	153 (2)
N4—H6⋯O3^i^	0.92 (2)	1.87 (2)	2.787 (1)	169 (2)
N4—H7⋯O2^ii^	0.90 (2)	1.91 (2)	2.808 (1)	175 (1)
N4—H8⋯O2	0.93 (2)	1.88 (2)	2.776 (1)	159 (2)
N4—H9⋯O1^iii^	0.91 (2)	2.42 (2)	3.068 (1)	128 (2)

Symmetry codes: (i) 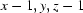; (ii) 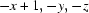; (iii) 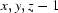.

**Fig. 1.**
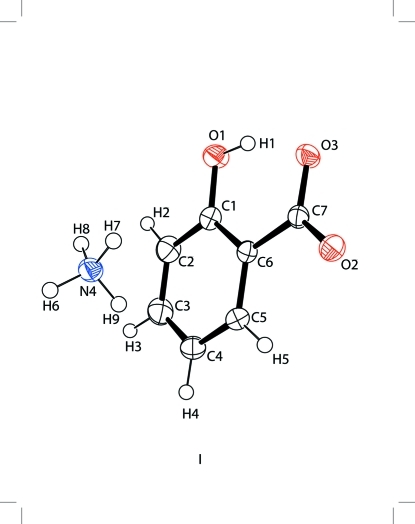
The molecular structure of (I) with labeling atoms. Displacement ellipsoids are drawn at the 50% probability level.

**Fig. 2.**
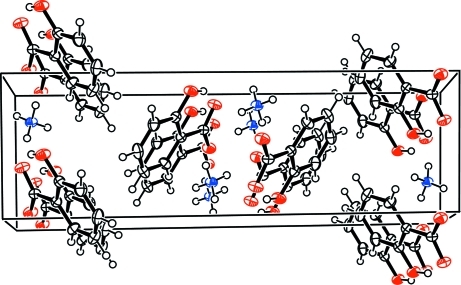
The crystal packing of (I) normal to (100).

**Fig. 3.**
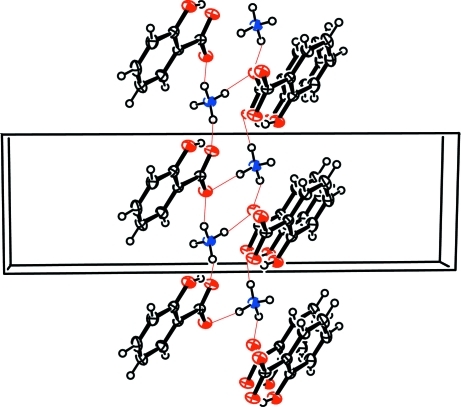
The hydrogen bonding (red lines) between ammonium and carboxylate ions in (I).

## supplementary crystallographic information

### Comment

There is an increasing demand for new materials that can be used for efficient, readily available, low-cost, high-energy neutron detection devices in the presence of a strong γ-ray background. The need for inexpensive neutron scintillators with reasonable optical transparency and fast response time led us to focus on growing, developing and characterizing single crystals of candidate materials. In this regard, materials based on organic scintillators are of particular interest because of their potential for low-level neutron detection *via* pulse shape discrimination (PSD) (Brooks, 1979; Kaschuck *et al.*, 2002; Kachuk & Esposito, 2005).

In search of new neutron detecting materials with enhanced performance (efficiency and cost), we considered materials that duplicate the structural features of commonly used materials, for example, salicylic acid is a common component in liquid scintillation systems. Salts of salicylic acid are good candidates for dry solid scintillators. Knowledge of these structural data is important to the development of a fundamental understanding of its scintillating properties, and more generally a predictive capability for tailoring materials to achieve desired scintillation properties. To address these challenges, we report here our measurements of the crystal structure of ammonium salicylate (I).

The asymmetric unit of (I) comprises a salicylate cation and an ammonium anion (Fig. 1). The projection of the unit cell contents on the *bc* plane is shown in Fig. 2. The hydrogen bonding between the carboxylate group and ammonium ions contributes to the stabilization of this crystal packing. This bonding allows two ammonium ions to connect two salicylate ions by forming alternating eight- and twelve- membered rings (Fig. 3). These alternating rings run as strips parallel to *c* axis and phenyl rings are outwardly attached to them in zigzag patterns. The phenyl rings are stacked along *a* axis. The oxygens of the hydroxyl groups form weak hydrogen bonding to the ammonium ions (see Table 1). Salicylate salts are not rare. Salicylic acid makes salts with not only ammonium but also alkali metals (Wiesbrock & Schmidbaur, 2003a, b; Dinnebier *et al.*, 2002).

Alkali salicylates are repoted as monohydrates (Li, Cs) (Wiesbrock & Schmidbaur, 2003a, b) or anhydrous forms (K, Rb) (Dinnebier *et al.*, 2002). Similar to (I), alkali salicylates also form double helix type ribbons with phenyl rings attached in zipper shapes. However, the difference lies in the linkage of the ribbons. Carboxylate groups, water molecules and metal ions form the ribbons of hydrated alkali salicylates whereas carboxylate groups, hydroxyl group and metal ions do those of anhydrous alkali salicylates. The connectivity forming ribbons in (I) is mainly through O···H—N hydrogen bonding between the carboxylate groups and ammonium ions (Table 1 and Fig. 3). The hydrogen bonding is comparable to that seen in other salicylate compounds (Gellert & Hsu, 1983; Drake *et al.*, 1993). In the case of Li, the phenyl rings are perpendicular to the ribbons. With larger alkali metals, the phenyl rings tilt toward the ribbons. (I) has weak hydrogen bonding between oxygens of hyddoxyl groups and ammonium ions, which favors tilt of the phenyl rings. In this type of structure, *π-π* stacking or hydrogen bonding between salicylate anions may not exist due to the large interplanar distance or co-planar distance between phenyl rings.

### Experimental

A repeated recrystallization process was applied. The crystals of (I) with high purity were obtained (1) from saturated commercial product (99%, Sigma-Aldrich)  from methanol solution or (2) by precipitation of a solution of salicylic acid (99% Sigma-Aldrich) and ammonium water. The single crystals were coated with paratone oil and mounted onto a cryo-loop pin.

### Refinement

Only non H-atoms were refined anisotropically.  H-atoms were found from difference Fourier and refined isotropically and freely, O-H = 0.93 (2) Å, range of N-H = 0.91 (2) to 0.933 (19) Å, and range of C-H = 0.952 (18) to 1.00 (2) Å.

### Crystal data

**Table:**

NH_4_^+^·C_7_H_5_O_3_^−^	*F*(000) = 328
*M**_r_* = 155.15	*D*_x_ = 1.366 Mg m^−^^3^
Monoclinic, *P*2_1_/*n*	Synchrotron radiation, λ = 0.77490 Å
Hall symbol: -P 2yn	Cell parameters from 3198 reflections
*a* = 6.0768 (6) Å	θ = 3.8–33.5°
*b* = 20.089 (2) Å	µ = 0.13 mm^−^^1^
*c* = 6.3353 (7) Å	*T* = 150 K
β = 102.768 (1)°	Plate, colorless
*V* = 754.28 (14) Å^3^	0.40 × 0.20 × 0.06 mm
*Z* = 4	

### Data collection

**Table:**

Bruker APEXII diffractometer	2274 independent reflections
Radiation source: 11.3.1 ALS, LBNL, CA	1939 reflections with *I* > 2σ(*I*)
Si (111)	*R*_int_ = 0.053
ω scans	θ_max_ = 33.8°, θ_min_ = 3.8°
Absorption correction: multi-scan (*SADABS*; Sheldrick, 2004)	*h* = −8→8
*T*_min_ = 0.950, *T*_max_ = 0.992	*k* = −27→28
7758 measured reflections	*l* = −9→8

### Refinement

**Table:**

Refinement on *F*^2^	Primary atom site location: structure-invariant direct methods
Least-squares matrix: full	Secondary atom site location: difference Fourier map
*R*[*F*^2^ > 2σ(*F*^2^)] = 0.049	Hydrogen site location: inferred from neighbouring sites
*wR*(*F*^2^) = 0.150	All H-atom parameters refined
*S* = 1.10	*w* = 1/[σ^2^(*F*_o_^2^) + (0.0888*P*)^2^ + 0.043*P*] where *P* = (*F*_o_^2^ + 2*F*_c_^2^)/3
2274 reflections	(Δ/σ)_max_ = 0.006
136 parameters	Δρ_max_ = 0.36 e Å^−^^3^
0 restraints	Δρ_min_ = −0.24 e Å^−^^3^

### Special details

**Table:**

Geometry. All e.s.d.'s (except the e.s.d. in the dihedral angle between two l.s. planes) are estimated using the full covariance matrix. The cell e.s.d.'s are taken into account individually in the estimation of e.s.d.'s in distances, angles and torsion angles; correlations between e.s.d.'s in cell parameters are only used when they are defined by crystal symmetry. An approximate (isotropic) treatment of cell e.s.d.'s is used for estimating e.s.d.'s involving l.s. planes.
Refinement. Refinement of *F*^2^ against ALL reflections. The weighted *R*-factor *wR* and goodness of fit *S* are based on *F*^2^, conventional *R*-factors *R* are based on *F*, with *F* set to zero for negative *F*^2^. The threshold expression of *F*^2^ > 2σ(*F*^2^) is used only for calculating *R*-factors(gt) *etc*. and is not relevant to the choice of reflections for refinement. *R*-factors based on *F*^2^ are statistically about twice as large as those based on *F*, and *R*- factors based on ALL data will be even larger.

### Fractional atomic coordinates and isotropic or equivalent isotropic displacement parameters (Å^2^)

**Table:**

	*x*	*y*	*z*	*U*_iso_*/*U*_eq_	
O1	0.75801 (14)	0.09351 (5)	0.40962 (13)	0.0303 (2)	
O2	0.24729 (14)	0.05282 (4)	0.06590 (12)	0.0292 (2)	
O3	1.12089 (13)	0.03981 (4)	0.36657 (12)	0.0283 (2)	
N4	0.47360 (16)	0.05050 (5)	−0.26977 (15)	0.0245 (2)	
C1	0.76231 (17)	0.12776 (5)	0.22639 (17)	0.0233 (2)	
C2	0.5922 (2)	0.17478 (6)	0.1553 (2)	0.0312 (3)	
C3	0.5876 (2)	0.21026 (6)	−0.0326 (2)	0.0347 (3)	
C4	0.7494 (2)	0.19945 (6)	−0.1538 (2)	0.0328 (3)	
C5	0.91906 (19)	0.15315 (5)	−0.08247 (18)	0.0273 (3)	
C6	0.92866 (16)	0.11662 (5)	0.10730 (16)	0.0211 (2)	
C7	1.11200 (16)	0.06636 (5)	0.18209 (15)	0.0217 (2)	
H1	0.884 (3)	0.0660 (9)	0.429 (3)	0.048 (5)*	
H2	0.482 (3)	0.1790 (8)	0.247 (2)	0.033 (4)*	
H3	0.461 (4)	0.2423 (10)	−0.088 (3)	0.061 (5)*	
H4	0.742 (3)	0.2241 (9)	−0.283 (3)	0.042 (4)*	
H5	1.036 (3)	0.1449 (8)	−0.165 (2)	0.036 (4)*	
H6	0.368 (3)	0.0433 (8)	−0.397 (3)	0.046 (5)*	
H7	0.563 (3)	0.0162 (8)	−0.212 (2)	0.038 (4)*	
H8	0.399 (3)	0.0624 (9)	−0.162 (3)	0.045 (4)*	
H9	0.563 (3)	0.0840 (10)	−0.297 (3)	0.051 (5)*	

### Atomic displacement parameters (Å^2^)

**Table:**

	*U*^11^	*U*^22^	*U*^33^	*U*^12^	*U*^13^	*U*^23^
O1	0.0288 (4)	0.0397 (5)	0.0258 (4)	0.0077 (3)	0.0131 (3)	0.0051 (3)
O2	0.0235 (4)	0.0393 (5)	0.0276 (4)	0.0052 (3)	0.0119 (3)	0.0037 (3)
O3	0.0232 (4)	0.0406 (5)	0.0217 (4)	0.0055 (3)	0.0063 (3)	0.0075 (3)
N4	0.0232 (4)	0.0297 (5)	0.0214 (4)	0.0017 (3)	0.0064 (3)	0.0013 (3)
C1	0.0224 (5)	0.0229 (5)	0.0251 (5)	−0.0007 (3)	0.0063 (4)	−0.0025 (3)
C2	0.0279 (5)	0.0271 (5)	0.0399 (6)	0.0057 (4)	0.0102 (5)	−0.0013 (4)
C3	0.0319 (6)	0.0226 (5)	0.0475 (7)	0.0041 (4)	0.0038 (5)	0.0056 (5)
C4	0.0310 (6)	0.0273 (5)	0.0383 (6)	−0.0021 (4)	0.0039 (5)	0.0117 (4)
C5	0.0247 (5)	0.0291 (5)	0.0280 (5)	−0.0030 (4)	0.0059 (4)	0.0060 (4)
C6	0.0187 (4)	0.0217 (4)	0.0223 (4)	−0.0018 (3)	0.0033 (3)	0.0000 (3)
C7	0.0179 (4)	0.0271 (5)	0.0200 (4)	−0.0004 (3)	0.0041 (3)	0.0004 (3)

### Geometric parameters (Å, °)

**Table:**

O1—C1	1.3547 (13)	C2—C3	1.3826 (18)
O1—H1	0.931 (19)	C2—H2	0.985 (17)
O2—C7^i^	1.2487 (13)	C3—C4	1.3915 (19)
O3—C7	1.2749 (12)	C3—H3	1.00 (2)
N4—H6	0.925 (18)	C4—C5	1.3878 (16)
N4—H7	0.902 (17)	C4—H4	0.952 (18)
N4—H8	0.933 (19)	C5—C6	1.3988 (14)
N4—H9	0.91 (2)	C5—H5	0.981 (17)
C1—C2	1.3991 (15)	C6—C7	1.5007 (14)
C1—C6	1.4065 (14)	C7—O2^ii^	1.2487 (13)
			
C1—O1—H1	104.0 (11)	C2—C3—H3	120.0 (12)
H6—N4—H7	118.3 (15)	C4—C3—H3	119.1 (12)
H6—N4—H8	108.8 (16)	C5—C4—C3	119.32 (11)
H7—N4—H8	104.2 (14)	C5—C4—H4	121.2 (11)
H6—N4—H9	106.2 (15)	C3—C4—H4	119.4 (11)
H7—N4—H9	108.3 (17)	C4—C5—C6	121.22 (11)
H8—N4—H9	111.1 (15)	C4—C5—H5	120.8 (9)
O1—C1—C2	117.78 (10)	C6—C5—H5	118.0 (9)
O1—C1—C6	122.07 (9)	C5—C6—C1	118.63 (9)
C2—C1—C6	120.14 (10)	C5—C6—C7	120.76 (9)
C3—C2—C1	119.88 (11)	C1—C6—C7	120.61 (9)
C3—C2—H2	125.3 (9)	O2^ii^—C7—O3	123.32 (9)
C1—C2—H2	114.8 (9)	O2^ii^—C7—C6	120.00 (9)
C2—C3—C4	120.81 (10)	O3—C7—C6	116.68 (9)
			
O1—C1—C2—C3	−179.03 (10)	C2—C1—C6—C5	−0.29 (15)
C6—C1—C2—C3	0.11 (17)	O1—C1—C6—C7	−0.95 (15)
C1—C2—C3—C4	0.53 (18)	C2—C1—C6—C7	179.95 (9)
C2—C3—C4—C5	−0.99 (18)	C5—C6—C7—O2^ii^	−5.84 (15)
C3—C4—C5—C6	0.81 (17)	C1—C6—C7—O2^ii^	173.93 (9)
C4—C5—C6—C1	−0.18 (16)	C5—C6—C7—O3	173.62 (9)
C4—C5—C6—C7	179.59 (10)	C1—C6—C7—O3	−6.62 (14)
O1—C1—C6—C5	178.82 (9)		

Symmetry codes: (i) *x*−1, *y*, *z*; (ii) *x*+1, *y*, *z*.

### Hydrogen-bond geometry (Å, °)

**Table:**

*D*—H···*A*	*D*—H	H···*A*	*D*···*A*	*D*—H···*A*
O1—H1···O3	0.93 (2)	1.66 (2)	2.523 (1)	153 (2)
N4—H6···O3^iii^	0.92 (2)	1.87 (2)	2.787 (1)	169 (2)
N4—H7···O2^iv^	0.90 (2)	1.91 (2)	2.808 (1)	175 (1)
N4—H8···O2	0.93 (2)	1.88 (2)	2.776 (1)	159 (2)
N4—H9···O1^v^	0.91 (2)	2.42 (2)	3.068 (1)	128 (2)

Symmetry codes: (iii) *x*−1, *y*, *z*−1; (iv) −*x*+1, −*y*, −*z*; (v) *x*, *y*, *z*−1.
